# Exploring Psychosocial Determinants of Eating Behavior: Fruit and Vegetable Intake Among Brazilian Adolescents

**DOI:** 10.3389/fnut.2021.796894

**Published:** 2021-12-16

**Authors:** César Henrique de Carvalho Moraes, Marle dos Santos Alvarenga, Jéssica Maria Muniz Moraes, Denise Cavallini Cyrillo

**Affiliations:** ^1^Graduate Program in Nutrition in Public Health, School of Public Health, University of São Paulo, São Paulo, Brazil; ^2^Department of Economics, School of Economics, Business and Accounting, University of São Paulo, São Paulo, Brazil

**Keywords:** eating behavior, adolescent, health diet, motivation, socioeconomic status, self-efficacy, social norms

## Abstract

In most Western countries, children and adolescents do not eat the recommended amount of fruits and vegetables (FVs). Theoretical frameworks on social psychology of eating, such as the Reason Action Approach, Social Cognitive Theory, and Theory of Normal Conduct have been applied to understand how psychosocial variables can explain FV intake. However, considering those predictors is still rare on the understanding of FV intake among adolescents (particularly in Brazil) despite its importance within eating behavior. Therefore, this study explored important psychosocial determinants of weekly frequency of FV intake among Brazilian adolescents in a model testing socioeconomic status (SES) and body mass index (BMI). A cross-sectional design was performed with 429 students (58% female), mean age 14.45 (SD 1.86). Key variables of theoretical framework on social psychology of food were investigated by structural equation modeling. The model included self-efficacy, attitudes, and social norms (with its subcomponents descriptive and injunctive) as psychosocial predictors of weekly frequency of FV intake along with SES and BMI. An instrument developed for Brazilian Portuguese was used to collect psychosocial variables as well as to verify FV reported intake. The total model explained 45.5% of weekly frequency of FV intake, and self-efficacy was the only significant psychosocial determinant (λ = 0.51, *p* = 0.001). SES also showed an important effect on the model (λ = 0.21, *p* = 0.001), while for BMI no significance was observed. In conclusion, the model was adequate to understand psychosocial determinants of weekly frequency of FV intake for Brazilian adolescents, with self-efficacy and SES as the major determinants of this eating behavior.

## Introduction

In most Western countries, children and adolescents eat far less fruit and vegetables (FV) than recommended (1). Among adolescents and young adults, 45% of individuals eat FV <5 times a week (2). Particularly in Brazil, 80% of adolescents eat inadequate amounts of this food group, and 30% do not eat any FV (3). Additionally, 90% of Brazilians ingest FV below the recommendations established by the Ministry of Health (400g/day) (4), a common situation since adolescence and which has become a public health concern in the country (5).

Many aspects can explain the lack of FV intake by adolescents, as eating behavior has multiple determinants. Among them, psychosocial determinants can have an important impact on FV, as they contribute to food preferences, the perception of what is healthy and to meaning and knowledge of food (6). Thus, knowing the relevance of key psychosocial determinants for adolescents in different contexts, and identifying the magnitude and which of these determinants have the greatest effect on FV intake are required. This knowledge can support initiatives for more assertive clinical practices, interventions, and recommendations aiming improvement of this class of eating behavior. Additionally, it is rare to find studies that evaluate sociodemographic variables (7, 8) and body mass index (BMI) (9) as moderators of psychosocial determinants of FV intake, even though consideration of these interactions is important (10, 11).

For those reasons, in a cross-sectional design, we aimed to (1) evaluate the relevance of psychosocial determinants, SES, and BMI as predictors of FV intake for Brazilian adolescents. Second, we aimed to (2) evaluate the magnitude of each determinant to FV intake, checking which one or ones are the most significant predictors.

### Theoretical Framework

When assessed, psychosocial determinants that potentially explain FV intake are evaluated within theoretical frameworks from social psychology (10, 12–15). Adults are the most studied group in the context of psychosocial determinants of FV intake through these frameworks, and few studies with adolescents are found (10). In Brazil, there is also a lack of studies investigating psychosocial determinants of FV intake among adolescents (16–21).

Social Cognitive Theory (22), the Normative Conduct Theory (23), the Reasoned Action Approach (24, 25), and strands, such as the Theory of Planned Behavior (26–29), are examples of frameworks that include psychosocial variables, such as self-efficacy, attitudes, and social norms (descriptive and injunctive) in models.

Self-efficacy, also called perceived behavioral control (30), includes individual's beliefs about abilities to overcome obstacles in the attempt to accomplish a behavior (22, 31). Attitudes refer to the beliefs arising from favorable or unfavorable evaluations that one makes about a goal, behavior, or another individual (32, 33). Social norms are a set of beliefs that emerge from environmental “cues” coming from a social group considered relevant to the person. Once perceived as social pressure, social norms lead to the adjustment of the individual to a social group considered relevant (34, 35). Descriptive social norms are characterized when individuals observe what the relevant social group around them does or thinks, while injunctive social norms refer to a perceived obligation imposed by the relevant group (36).

These factors are assessed together in various ways in studies that aimed to investigate psychosocial determinants for FV intake in adults and young adults (10, 13, 15, 37–40). For adolescents, these determinants were evaluated in three studies: one of them through focus groups (41) and two by non-validated instruments (11, 42). A meta-analysis (25) shows that social norms are rarely considered in their descriptive and injunctive subcomponents in health behavior scenarios. To our knowledge, there are three studies considering the subcomponents (11, 37, 43), two of which refer to FV intake (11, 37). Of these, only one study evaluated adolescents (11) and none of them used a validated instrument. In Brazil, we found only one study that evaluated psychosocial determinants of eating behavior according to social psychology theories, but with focus on fish intake and no exclusive evaluation among adolescents considering SES and BMI as moderators (44).

**Figure 1:**
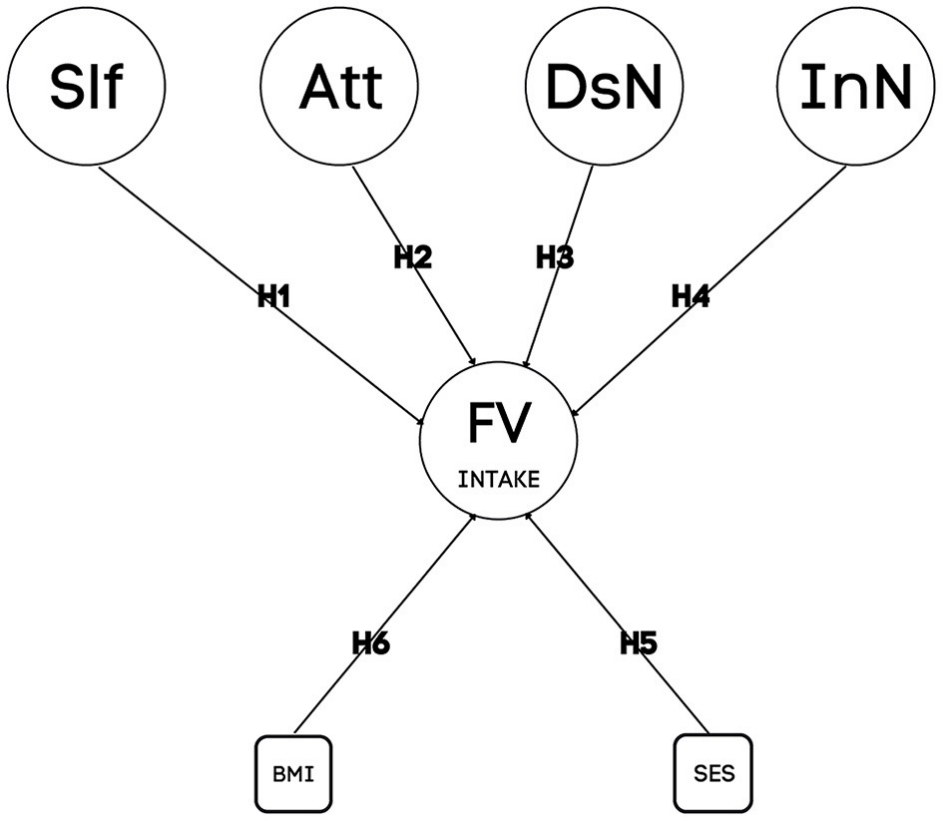
Proposed prediction model with hypotheses for weekly frequency of Fruit and Vegetable Intake regarding social psychological determinants, Socioeconomic Status (SES), and Body Mass index (BMI). H1–H6, Hypothesis 1–6; Sl, Self-efficacy; Att, Attitudes; DsN, Descriptive social norms; InN, Injunctive social norms.

Because of that, we tested the hypotheses that weekly frequency of FV intake among adolescents is explained by self-efficacy (Hypothesis 1); attitudes (Hypothesis 2); descriptive social norms (Hypothesis 3); injunctive social norms; (Hypothesis 4); socioeconomic status (Hypothesis 5); and body mass index (Hypothesis 6). The model of our hypotheses is illustrated in Figure 1.

## Materials and Methods

### Participants

A cross-sectional study was conducted with a sample of adolescents of both sexes recruited from six middle and high schools in three cities in the state of São Paulo, Southeast Brazil. Eligible participants were between 10 and 19 years old, without intellectual disabilities as identified by teachers. Both the participants and their caregivers signed the informed consent form. A non-probability-type sample was used. Sample size was defined based on Forero et al. (45) who recommend 200 to 500 cases to studies using *Factor Analyses with ordinal indicators*.

### Measures

Sex, age, level of education of adolescent and respective caregiver, and skin color were self-reported. SES was evaluated by the number of items present in the individual's daily life, based on a questionnaire from the Brazilian Association of Business and Research (46). The items were bathroom, computer, microwave oven, refrigerator, freezer, washing machine, dishwasher, dryer, and monthly cleaning assistant (responses from “none” to “2 or more items”).

Self-reported weight and height were considered equivalent to the measurement versions (46). BMI was calculated by dividing each participant's weight in kg by height in square meters (m).

The refined instrument *Psychosocial Influences for fruit and vegetable Eating Scale* (PSI-FAVES)–*submitted manuscript*–evaluated the frequency of weekly FV intake reported and its psychosocial determinants for adolescents. The instrument is composed of 28 items distributed in five factors: (1) Weekly frequency of FV intake (three items) with responses from 1 (never) to 8 (7 times a week); (2) Self-efficacy (eight items) ranging from 1 (not sure) to 5 (completely sure); (3) Attitudes (nine items) ranging from 1 (strongly disagree) to 5 (strongly agree); (4) Descriptive social norms (four items) ranging from 0 (don't know) to 5 (strongly agree); and 5. Injunctive social norms (four items) ranging from 1 (do not know) to 5 (strongly agree). The complete instrument can be found in Table 2 of Supplementary Material.

The predictive model proposed (Figure 1) considered as dependent variable the frequency of weekly FV intake. Independent variables were self-efficacy, attitudes, descriptive social norms, injunctive social norms, SES, and BMI.

### Procedures

The participants answered an online survey in their schools directly from computer rooms in each site. The computer rooms were prepared in advance with a link to access the set of survey instruments. The link was generated by the REDCap data management system (47), which stored the instruments and collected data securely simultaneously and online.

### Data Analysis

The analyses were conducted using R (48) and JASP (49). Descriptive statistics were performed to characterize the sample. The predictive mean matching (PMM) technique was used for problems with missing data (50). Missing data frequencies below 5% were considered irrelevant (51). The significance level adopted for all analyses was *p* < 0.05. The distribution of items in each factor and the SES and BMI variables were tested for multivariate normality through skewness and kurtosis. Data normality was verified with values between −2 and 2 for skewness, and between −7 and 7 for kurtosis (52). Descriptive statistics were calculated by means and standard deviation for continuous variables, and frequency and percentages for categorical variables.

### FV Intake Prediction Model

Descriptive statistics (means and standard deviation) of PSI-FAVES are presented in **Table 2**. All multivariate analyses were performed by diagonally weighted least squares (DWLS) estimation (53). First, the adjustment of PSI-FAVES to the sample was presented through a measurement model by confirmatory factor analysis (CFA), while a structural equation model (SEM) was used to verify the psychosocial variables, SES, and BMI as predictors of weekly frequency of FV. For both CFA and SEM, results indicate acceptable model fit when values of Comparative Fit Index (CFI) and Tucker-Lewis Index (TLI) are ≥ 0.9; Root Mean Square Error of Approximation (RMSEA) for a 90% Confidence Interval (CI) is ≤ 0.08 and Standardized Root Mean Residual (SRMR) is ≤ 0.08 (52–54).

For the measurement model from PSI-FAVES, convergent validity was calculated using the average extracted variance (AVE). Factors with values of AVE ≥0.5 indicated adequate convergent validity (55). The reliability per items and factors, and the total reliability of PSY-FAVES were calculated by McDonald's omega coefficient (ω). With a range between zero and one, the higher the values of ω, the higher the reliability indicator (56). Factors that had AVE values ≤ 0.5 but reliability ≥0.6 were still considered adequate for convergent validity (57). Discriminant validity was calculated by hetero-trait mono-trait (HTMT) analysis. Values of HTMT ≤ 0.9 indicated adequate discriminant validity (58).

The *R*-squared (*R*^2^) coefficient of determination was used to measure the explained variance of the full model. Once these quality criteria were met, it was possible to test our hypotheses. To this end, the factor loadings (λ) of the determinants were considered as effect sizes and they were compared to each other to verify which had the greatest relative weights in determining the weekly frequency of FV intake (53). From zero to one, values of λ ≥ 0.4 were considered acceptable. From that, the higher the value of λ, the more significant was the determinant (52).

## Results

### Participants and Characteristics

The sample consisted of 429 participants (58% women), with mean age of 14.5 (SD 1.88) (women = 14.4, SD 2.13; men = 14.5, SD 1.82). The mean BMI (Kg/m^2^) was 21.37 (SD 3.97). The mean of socioeconomic items used to estimate socioeconomic status was 8 items (SD 0.23). The participants self-identified as white (52.2%), brown (36.5%), black (8%), yellow (2.6%), and indigenous (0.7%). The sample characteristics are described in Table 1.

**Table 1:** Sample characteristics.

	***N* (%)**	**Mean (SD)**	**Range**
Sex^*^			
Female	249 (58.0%)		
Male	180 (42.0%)		
Age (in years)		14.45 (1.86)	10.91–19.33
BMI (kg/m^2^)		21.48 (4.14)	13.21–38.89
SES^a^		0.79 (0.23)	0.00–2.00
Adolescents' caregiver^*^			
Mother/father	395 (92.07%)		
Grandmother/grandfather	25 (5.82%)		
Partner	1 (0.23%)		
Uncle/aunt	4 (0.93%)		
Him/Herself	3 (0.69%)		
Another person	1 (0.23%)		
Caregiver's level of education^**^			
Incomplete elementary School	25 (5.82%)		
Elementary School or Grade School	36 (8.40%)		
Middle School	104 (24.29%)		
High School	113 (26.34%)		
University education	120 (27.97%)		
Postgraduate	31 (7.23%)		
Ethnicity^*^			
White	222 (51.74%)		
Mixed race	157 (36.59%)		
Black	36 (8.39%)		
Japanese	11 (2.56%)		
Indigenous	3 (0.69%)		

a *Obtained by the average number of socioeconomic items (bathroom, computer, microwave, oven, refrigerator, freezer, washing machine, dishwasher, clothes dryer, housekeeper, car, motorcycle, and DVD)*.

* *Missing values lower than 1%*.

** *Missing values lower than 3%*.

All parameters showed normal distribution considering the properties of skewness and kurtosis (Table 1 of Supplementary Material). PSY-FAVES showed adequate adjustment to the sample [CFI = 0.96; TLI = 0.95; RMSEA (90% CI) = 0.043 (0.038–0.049); SRMR = 0.066]. Convergent validity was observed for FV (AVE = 0.78; ω = 0.52), self-efficacy (AVE = 0.57; ω = 0.83), attitudes (AVE = 0.54; ω = 0.82), and injunctive norms (AVE = 0.45; ω = 0.64). Convergent validity concerns were observed for descriptive norms (AVE = 0.3; ω = 0.52). As for discriminant validity, all values were adequate for all factors (HTMT = 0.06–0.65) (Tables 3, 4 of Supplementary Material). All factor loadings (λ) of items of the instrument were adequate (λ = 0.42–0.96). The descriptive statistics, fit indices, reliability, factor loadings, and convergent validity of PSY-FAVES are presented in Table 2.

**Table 2:** Descriptive statistics, McDonald's omega coefficient (for the whole instrument, items and their factors), factor loadings (λ), and average variance extracted (AVE) of the *Psychosocial Influences for fruit and vegetable Eating Scale* (PSY-FAVES)^a^.

**Factor and determinants**	**Mean (SD)**	**McDonald's omega (ω)**	**λ**	**SE**	***p*-value**	**AVE^**d**^**
		ω total (PSY-FAVES) = 0.86				
**FV** ^**b**^ ^.^ ^**c**^						
Fr1: “*How many times do you eat fruits in your breakfast”*	1.29 (1.65)	0.32	0.79	0.08	***	0.78
Fr2: “*How many times do you eat fruits in your lunch”*	1.32 (1.70)	0.47	0.91	0.09	***	
Fr3: “*how many times do you eat vegetables in your intervals (“e.g., sandwich with thin carrots. lettuce or tomatoes for a morning or afternoon snack)”*	1.76 (1.93)	0.45	0.96	0.09	***	
		ω (factor): 0.52				
**Self-efficacy (Slf)**						
Self1: “*I feel able to eat more fruit and vegetables everyday”*	3.65 (1.18)	0.80	0.74	0.03	***	0.57
Self2: “*I feel able to eat more fruit and vegetables when I get home from school or work”*	3.41 (1.17)	0.81	0.77	0.03	***	
Self3: “*I feel able to eat more fruit and vegetables while watching TV”*	3.17 (1.27)	0.81	0.79	0.03	***	
Self4: “*I feel able to eat more fruit and vegetable while using computer or cellphone”*	2.88 (1.32)	0.82	0.70	0.03	***	
Self5: “*I feel able to eat fruit and vegetable when my friends are around”*	2.88 (1.25)	0.88	0.88	0.03	***	
Self6: “*I feel able to eat fruit and vegetable when I'm bored”*	2.38 (1.22)	0.81	0.70	0.03	***	
Self7: “*I feel able to eat fruit and vegetable when I'm in a bad mood”*	2.38 (1.19)	0.81	0.73	0.03	***	
Self8: “I feel able to eat fruit and vegetables when I'm busy”	2.70 (1.26)	0.82	0.72	0.03	***	
		ω (factor): 0.83				
**Attitudes (Att)**						
At1: “*If I eat fruits and vegetables I'll like myself better”*	3.22 (1.31)	0.79	0.90	0.04	***	0.54
At2: “*If I eat fruit and vegetables I'll lose weight”*	3.66 (1.21)	0.82	0.46	0.03	***	
At3: “*If I eat fruits and vegetables I'll look better (e.g., my skin. hair and nails will look better)”*	4.09 (1.08)	0.81	0.52	0.03	***	
At4: “*If I eat fruits and vegetables I'll look better (e.g., my skin. hair and nails will look better) and I'll be more confident with myself when my friends are around”*	3.62 (1.24)	0.79	0.79	0.03	***	
At5: “*If I eat fruits and vegetables I will look better (e.g., my skin. hair and nails will look better) and I would like to show this improvement by posting texts and/or photos on social media (Facebook®. Instagram®. Periscope®. Snapchat®. Blogs. Etc.)*	3.03 (1.41)	0.80	0.76	0.04	***	
At6: “*If I eat more fruits and vegetables my family will also eat more”*	3.26 (1.28)	0.80	0.75	0.03	***	
At7: “*If I eat more fruits and vegetables my family will be proud of me”*	3.66 (1.23)	0.80	0.75	0.03	***	
At8: “*If I eat more fruits and vegetables. I will also influence my friends to eat more”*	3.04 (1.30)	0.80	0.86	0.04	***	
At9: “*If I eat more fruits and vegetables. I will be an example of health to my friends”*	3.61 (1.25)	0.81	0.70	0.03	***	
		ω (factor): 0.82				
**Descriptive norms (DsN)**						
Descriptive1: “*My mother eats fruits and vegetables”*	4.28 (0.96)	0.44	0.42	0.04	***	0.30
Descriptive2: “M*y father eats fruits and vegetables”*	3.86 (1.28)	0.48	0.58	0.05	***	
Descriptive3: “M*y friends eat fruits and vegetables”*	2.96 (1.12)	0.50	0.61	0.05	***	
Descriptive4: “*People I follow on social media (Facebook. Instagram. Periscope. Snapchat. Blogs) post photos eating fruits and vegetables because they seem to care about their health”*	2.63 (1.37)	0.58	0.55	0.05	***	
		ω (factor): 0.52				
**Injunctive norms (InN)**						
Injunctive1: “*My mother thinks I should eat more fruits and vegetables”*	4.24 (1.06)	0.53	0.45	0.03	***	0.45
Injunctive2: “*My father thinks I should eat more fruits and vegetables”*	3.95 (1.26)	0.54	0.58	0.04	***	
Injunctive3: “*My friends think I should eat more fruits and vegetables”*	2.59 (1.27)	0.86	0.86	0.05	***	
Injunctive4: “*People I follow on social networks (Facebook. Instagram. Periscope. Snapchat. Blogs) argue that healthy habits like eating fruits and vegetables are important things and that's why I think I should think and do the same”*	3.27 (1.34)	0.76	0.76	0.05	***	
		ω (factor): 0.64				

a *Based on Confirmatory Factor Analysis (CFA) for a measurement model. Fit indices: Comparative Fit Index (CFI) = 0.96; Tucker-Lewis Index (TLI) = 0.95; Root Mean Square Error of Approximation (RMSEA) = 0.043 (90% Confidence Interval = 0.038–0.049); Standardized Root Mean Square Residual (SRMR) = 0.066*.

b *The weekly frequency was measure by the item: “Thinking in an ordinary week” following by the frequency options for each situation*.

c *Missing values lower than 3%*.

d *Calculated by the sum squares of factors loadings from CFA divided by the number of indicators of each factor*.

***p < 0.001.

### FV Intake Prediction Model Using SEM

The SEM showed adequate fit of the model to the data, with CFI = 0.94, TLI = 0.93, RSMEA = 0.047 (90% CI = 0.045–0.053), and SRMR = 0.068. The full model had an explanatory power of 45.5% (*R*^2^ = 0.455) (Table 3).

**Table 3:** Goodness-of-fit, explained variance, and factor loadings for the prediction model to weekly frequency of fruit and vegetable (FV) intake^a^.

**CFI = 0.94, TLI = 0.93, RMSEA = 0.047 (90% CI = 0.042–0.052), SRMR = 0.068**					
***R***^**2**^ **(whole model) = 0.455**					
**Factor**		**Determinant**	**λ**	**SE**	***p*-value**
FV	→	Fr1	0.47	0.00	-^a^
FV	→	Fr2	0.52	0.13	***
FV	→	Fr3	0.51	0.14	***
Self-efficacy	→	Self1	0.63	0.00	–^a^
Self-efficacy	→	Self2	0.66	0.06	***
Self-efficacy	→	Self3	0.62	0.07	***
Self-efficacy	→	Self4	0.54	0.06	***
Self-efficacy	→	Self5	0.70	0.07	***
Self-efficacy	→	Self6	0.57	0.060	***
Self-efficacy	→	Self7	0.62	0.06	***
Self-efficacy	→	Self8	0.57	0.06	***
Atittudes	→	At1	0.68	0.00	***
Atittudes	→	At2	0.38	0.04	***
Atittudes	→	At3	0.48	0.04	***
Atittudes	→	At4	0.64	0.05	***
Atittudes	→	At5	0.54	0.05	***
Atittudes	→	At6	0.59	0.05	***
Atittudes	→	At7	0.60	0.05	***
Atittudes	→	At8	0.67	0.06	***
Atittudes	→	At9	0.56	0.05	***
DescritiveNorms	→	DsN1	0.44	0.00	–^a^
DescritiveNorms	→	DsN2	0.45	0.14	***
DescritiveNorms	→	DsN3	0.55	0.14	***
DescritiveNorms	→	DsN4	0.40	0.15	***
InjuntiveNorms	→	InN1	0.42	0.00	–^a^
InjuntiveNorms	→	InN2	0.46	0.13	***
InjuntiveNorms	→	InN3	0.67	0.17	***
InjuntiveNorms	→	InN4	0.56	0.16	***
FV	←	Self-efficacy (Slf)	0.51	0.12	***
FV	←	Atittudes (Att)	0.17	0.13	0.24
FV	←	DescritiveNorms (DsN)	0.27	0.38	0.18
FV	←	InjuntiveNorms (InN)	−0.39	0.42	0.10
FV	←	BMI	−0.01	0.01	0.91
FV	←	SES	0.21	0.20	***

a *Based on Structural Equation Modeling (SEM) for a structural model. CFI, Comparative Fit Index; TLI, Tucker-Lewis Index; RMSEA, Root Mean Square Error of Approximation [90% Confidence Interval (CI)]; SRMR, Standardized Root Mean Square Residual; BMI, Body Mass Index; SES, Socioeconomic Status*;

***p < 0.001.

*^a^Dashed lines represent non-standardized estimates that were constraint to one as reference to the scale*.

Self-efficacy was the only significant determinant of weekly frequency of FV intake corroborating prior hypothesis 1. Hypotheses 2, 3, and 4 were not confirmed in our sample and the less significant psychosocial determinant was attitudes. SES was a significant determinant for the model (confirming hypothesis 5), and no effect of BMI was found (rejecting hypothesis 6).

**Figure 2:**
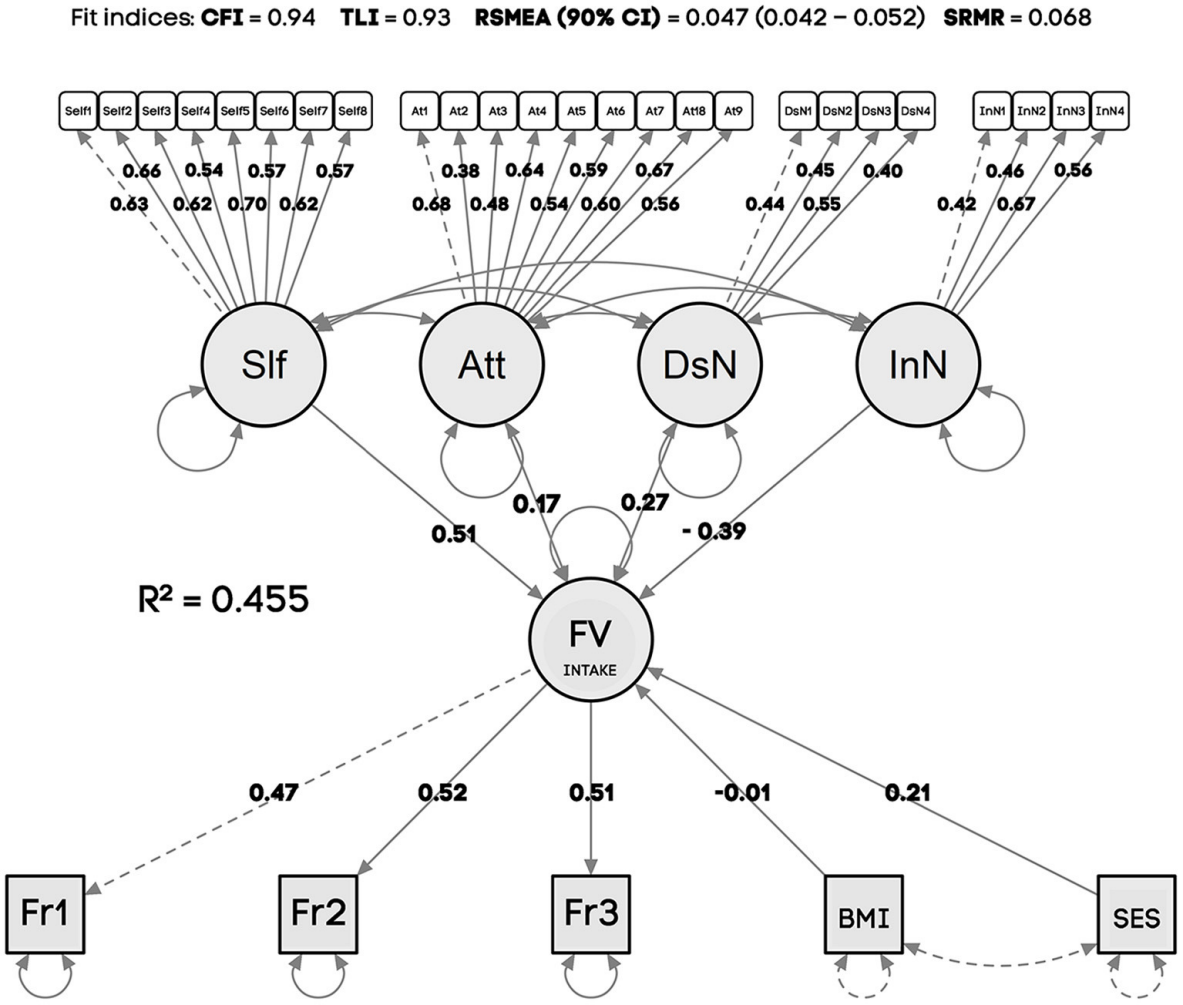
Prediction model for weekly frequency of Fruit and Vegetable Intake regarding psychosocial determinants, SES, and BMI. R-Square (*R*2), Variance explained by the model; CFI, Comparative Fit Index; TLI, Tucker-Lewis Index; RMSEA, Root Mean Square Error of Approximation [90% confidence interval (CI)]; SRMR, Standardized root mean square residuals; BMI, Body Mass Index; SES, Socioeconomic Status. Numbers in bold: factor loadings. Dashed lines represent non-standardized estimates that were constraint to one as reference to the scale.

By analyzing the items that determined each factor (Table 3), weekly frequency of FV intake, self-efficacy (Slf), descriptive social norms (DsN), injunctions (InN), and attitudes (Att), we found that the most significant determinants of FV intake were Fr2: “How many times do you eat fruits in your lunch?” and Fr3: “how many times do you eat vegetables in your intervals (“e.g., sandwich with thin carrots. lettuce or tomatoes for a morning or afternoon snack).” The most significant determinants of self-efficacy (Slf) were Sefl5: “I feel able to eat fruit and vegetable when my friends are around” and Self2: “I feel able to eat more fruit and vegetables when I get home from school or work.” Despite being not significant as a determinant of FV intake, attitudes had as most relevant predictors At1: “If I eat fruits and vegetables, I'll like myself better” and At: “If I eat more fruits and vegetables. I will also influence my friends to eat more.” Descriptive social norms (DsN) had as main determinants DsN3: “My friends eats fruits and vegetables,” DsN2: “My father eats fruits and vegetables,” and DsN1: “My mother eats fruits and vegetables.” Injunctive social norms (InN), which showed a negative non-significant relationship with FV intake, had as most expressive predictors InN3: “My friends think I should eat more fruits and vegetables” and InN4: “People I follow on social networks (Facebook, Instagram, Periscope, Snapchat, Blogs) argue that healthy habits like eating fruits and vegetables are important things and that's why I think I should think and do the same.” The complete predictive model is shown in the diagram presented in Figure 2.

## Discussion

This study explored key psychosocial determinants (self-efficacy, attitudes, descriptive social norms, and injunctive social norms), along with potential moderators SES and BMI, to understand the weekly frequency of FV intake among Brazilian adolescents. A prediction model to test the relationship of these variables was elaborated. The model showed adequate predictive capacity and indicated that among psychosocial determinants, self-efficacy was the only one that significantly determined FV intake. Additionally, SES showed a significant effect on the model, while BMI showed no effect on the model.

### Theoretical Implications of FV Intake Prediction Model

There is limited evaluation of the psychosocial determinants of FV intake among adolescents (11, 42), and to our knowledge, there is none in Brazil. Most of the studies were conducted among adults [e.g., (10, 15, 39)], and previous findings with this group have indicated that the explained variance by psychosocial determinants for FV intake in multivariate models range from 11 to 68% (0.11 < *R*^2^ < 0.68) [e.g., (10, 39, 54)]. For adolescents, a study that evaluated these determinants using a multivariate model found that the explained variance of the model was 45% (*R*^2^ = 0.45) (38). The explained variance in the model of our study was very similar to that: 45.5% (*R*^2^ = 0.455).

We had an issue with the convergent validity for descriptive social norms. This may indicate that descriptive social norms were not equally consistent for our sample. Thus, there may be divergence regarding which reference group (father, mother, friends, or social media) is most relevant to the adolescents. Similar reliability issues were found by Pedersen et al. (11). Although the authors did not present convergent validity analyses, low reliability for descriptive social norms for the Danish adolescents was also found. They suggest that despite this norm being not equally coherent, it is still reasonable to assume that it exists (11).

### Practical Implications of FV Intake Prediction Model

In our study, among the relevant contexts for our sample, the weekly frequency of FV intake came from fruits eaten during main lunch meals (i.e., “How many times do you eat fruits in your lunch”), breakfast (i.e., “How many times do you eat fruits in your breakfast”); and vegetables in the intervals [i.e., “How many times do you eat vegetable in your intervals” (e.g., sandwich with thin carrots, lettuce or tomatoes for a morning or afternoon snack)]. One possible explanation why the frequency of fruit during lunch was the strongest predictor of FV intake is that the sample was from public schools that provide fruits as part of school lunch menu. No other data of this nature were found for adolescents and in Brazil. Leal et al. investigated the pattern of FV intake of adolescents (59), but there was no focus on which of the meals these foods were most frequently eaten.

Regarding psychosocial determinants, with few exceptions (14, 39, 57), self-efficacy is shown to be the most relevant determinant of intentions to eat FV by adults [e.g., (10, 15, 58)], which is not always the case when self-efficacy is assessed as a direct determinant of FV intake (12, 15, 60). Of the two studies that quantitatively assessed psychosocial determinants for adolescents' FV intake (11, 42), one found a higher correlation between daily intake and self-efficacy (*r* = 0.373, *p* < 0.001) (42), while the other found greater relevance of self-efficacy directly on FV intake in a multivariate analysis (λ = 0.39, *p* < 0.001) (11).

In our study, the most relevant predictor of self-efficacy was Self5: “I feel able to eat fruit and vegetable when my friends are around.” Thus, our data agree with theories of social behavior, which describes that the influence of parents on adolescents gradually decreases throughout this phase, and that the influence of friends predominates (61). Notably, even though the psychosocial determinants descriptive and injunctive social norms and attitudes were not significant for adolescents' FV intake, we found that the reference group *friends* was always present among the most significant predictors of each factor. However, in our sample, friends were more relevant as encouragers of adolescents' sense of their abilities to eat FV (i.e., self-efficacy).

Self2: “I feel able to eat more fruit and vegetables when I get home from school or work” and Self3: “I feel able to eat more fruit and vegetables while watching TV” were the next most relevant predictors for self-efficacy. In fact, adolescent's home may be a place where they may express fewer barriers to eat fruit, as parents and caregivers become the reference in the absence of friends. In addition, fruits may be more readily available at home, which may provide adolescents greater insight into their ability to eat them. Previous data with American adolescents verify significant effects of self-efficacy evaluated by one question (i.e., “I feel confident in my ability to eat fruits and vegetables every day”) and perceived availability of FV over fruit and vegetables intake. Regarding self-efficacy to eat fruits and vegetables while watching TV, our data indicated an inverse relationship between TV-watching time and FV intake (62, 63). The television show content watched by adolescents should be assessed, as different contents can elicit different patterns of food intake (64).

Our study also showed a significant effect of SES on the weekly frequency of FV intake among adolescents, which highlights the importance of routinely testing such variables in studies looking at determinants of FV intake, including those investigating psychosocial determinants. It is not common in studies of this nature to assess variables pertaining to context such as life experience or sociodemographic variables (10), but it is known that socioeconomic variables play a crucial role in food preferences and may present themselves as barriers to the adoption of a healthy eating behavior (65) or to be positively associated with FV intake by adolescents and children (5). The absence of this evaluation in other surveys does not allow us to understand if the finding is particular to a country with more serious socioeconomic issues, like Brazil, or if it is also valid for more developed countries, which can also be explored in future studies.

The lack of relevance of BMI for the weekly frequency of FV intake in our findings may be justified by the fact that a single analysis of weekly frequency FV intake is very specific data. Additionally, it is known that changes in weight and nutritional status result from a complex interaction of factors (such as genetic, metabolic, and behavioral) and a great impact of environmental factors on the prevalence of obesity (66), which makes it unreasonable to justify BMI only by the weekly frequency of FV intake. This fact also highlights the need discuss obesity not only because of individual choices (67, 68).

Some limitations in this study need to be highlighted. First, all the data were cross-sectional, and although our objective was not to propose a definitive predictive model according to psychosocial variables, caution is needed in interpretation of the results. Additionally, the participants provide a self-report on weekly frequency of FV intake, which does not represent necessarily the actual intake. Another aspect is that the participants knew that the researcher was a dietitian, which may have influenced the results. The use of scales may also bring limitations because of the restricted possibility of responses or natural biases from self-report measures. Social acceptance may underlie these biases. Finally, regarding the evaluation of SES, the option to evaluate this aspect through a scale ranging only from “0 to 2 items or more” may have modified the magnitude of the effect of this variable on the weekly frequency of FV intake.

Despite the limitations, as far as we know, this study is the first to explore psychosocial determinant FV intake among adolescents using a validated instrument that investigates the descriptive and injunctive subcomponents of social norms in a model with two intervening variables: SES and BMI. Thus, our findings open the opportunity for more studies to investigate and identify which of these variables show more significance for adolescents in different contexts. Interventions aimed at promoting healthy eating encounter many barriers (5, 69) starting with communication strategies that routinely focus only on the benefit of consuming FV and do not generate improved intake when compared to alternative strategies (70–72). Thus, better understanding of factors related to FV intake for different groups can bring more effective actions, such as reinforcing skills and reducing barriers for FV intake (as suggested by self-efficacy as a relevant element in our sample). That situation was already verified in an experiment that evoked self-efficacy beliefs to encourage switching from energy-dense foods to fruits and vegetables among adolescents (73). Considering the reference group “friends” in the communication approach with adolescents is also an important strategy to encourage FV intake because this social group was the most relevant in the evaluation of the various psychosocial determinants investigated.

Future research should focus more on experimental models to test the effects of each one of the psychosocial determinants of FV intake among adolescents in more controlled settings. They may also include other variables of interest, such as body image, media influence, and the comparison of diverse sociocultural contexts.

## Conclusion

This research found that among the key psychosocial determinants of FV intake among adolescents, self-efficacy was the only significant one for our sample. Our findings suggest that psychosocial variables and socioeconomic variables should be in the routine of adolescents' eating behavior evaluation. Finally, this research calls for more studies to assess different kinds of adolescents from different regions and backgrounds to increase data that will support more accurate interventions aimed at increasing weekly frequency of FV intake. Through this knowledge-based communication of psychosocial determinants, emphasizing those determinants that already influence adolescents (e.g., self-efficacy) and increasing the relevance of those that are not significantly influential to the group (e.g., descriptive social norms or attitudes) can be fundamental tools to increase FV intake.

## Data Availability Statement

The original contributions presented in the study are included in the article/Supplementary Materials, further inquiries can be directed to the corresponding author.

## Ethics Statement

The research was approved by the Ethics Committee of the Faculty of Pharmaceutical Sciences of the University of São Paulo under registered number 1.919.946. Written informed consent to participate in this study was provided by the participants' legal guardian/next of kin.

## Author Contributions

CM was the principal investigator responsible for the study. CM and DC planned the design and made a substantial contribution to the editing of the manuscript. CM defined and ran the statistical analysis, established the size of the sample, and collected the data for the study. MA contributed greatly to the drafting of the manuscript. MA, JM, and DC read and approved the final version of the manuscript. All authors contributed to the article and approved the submitted version.

## Funding

The project was supported by the National Council for Scientific and Technological Development (CNPq) through a master's scholarship (CNPq, process 134136/2015-2).

## Conflict of Interest

The authors declare that the research was conducted in the absence of any commercial or financial relationships that could be construed as a potential conflict of interest.

## Publisher's Note

All claims expressed in this article are solely those of the authors and do not necessarily represent those of their affiliated organizations, or those of the publisher, the editors and the reviewers. Any product that may be evaluated in this article, or claim that may be made by its manufacturer, is not guaranteed or endorsed by the publisher.

## References

[1] Beal T Morris SS Tumilowicz A. Global patterns of adolescent fruit, vegetable, carbonated soft drink, and fast-food consumption: a meta-analysis of global school-based student health surveys. Food Nutr Bull. (2019) 40:444–59. 10.1177/0379572119848287 31617415

[2] INCA. Inquérito Domiciliar sobre Comportamentos de Risco e Morbidade Referida de Doenças e Agravos Não Transmissíveis. Rio de Janeiro: INCA (2003). Available online at: http://www.inca.gov.br/publicacoes/publicacao_inquerito22_06.pdf (accessed October 10, 2021).

[3] Machado RHV Feferbaum R Leone C. Consumo de frutas no Brasil e prevalência de obesidade. Rev Bras crescimento e Desenvolv Hum. (2016) 26:243–52. 10.7322/jhgd.119293

[4] IBGE. POF 2008-2009: mais de 90% da população comem poucas frutas, legumes e verduras. Brasília: IBGE (2011). Available online at: https://agenciadenoticias.ibge.gov.br/agencia-sala-de-imprensa/2013-agencia-de-noticias/releases/14059-asi-pof-2008-2009-mais-de-90-da-populacao-comem-poucas-frutas-legumes-e-verduras (accessed October 10, 2021).

[5] Rasmussen M Krølner R Klepp K-I Lytle L Brug J Bere E. Determinants of fruit and vegetable consumption among children and adolescents: a review of the literature. Part I: Quantitative studies. Int J Behav Nutr Phys Act. (2006) 3:22. 10.1186/1479-5868-3-22 16904006 PMC1564033

[6] Story M Neumark-Sztainer D French S. Individual and environmental influences on adolescent eating behaviors. J Am Diet Assoc. (2002) 102:S40–51. 10.1016/S0002-8223(02)90421-9 11902388

[7] Van Lenthe FJ Jansen T Kamphuis CBM. Understanding socio-economic inequalities in food choice behaviour: can Maslow's pyramid help? Br J Nutr. (2015) 113:1139–47. 10.1017/S0007114515000288 25784199

[8] Li ASW Figg G Schüz B. Socioeconomic status and the prediction of health promoting dietary behaviours: a systematic review and meta-analysis based on the theory of planned behaviour. Appl Psychol Heal Well-Being. (2019) 11:382–406. 10.1111/aphw.12154 30884154

[9] Caso D Capasso M Fabbricatore R Conner M. Unhealthy eating and academic stress: the moderating effect of eating style and BMI. Heal Psychol Open. (2020) 7:2055102920975274. 10.1177/2055102920975274 33294205 PMC7708726

[10] Guillaumie L Godin G. Psychosocial determinants of fruit and vegetable intake in adult population: a systematic review. Int J Behav Nutr Phys Act. (2010) 7:12. 10.1186/1479-5868-7-12 20181070 PMC2831029

[11] Pedersen S Grønhøj A Thøgersen J. Following family or friends. Social norms in adolescent healthy eating. Appetite. (2014) 86:54–60. 10.1016/j.appet.2014.07.030 25088047

[12] Povey R Conner M Sparks P James R Shepherd R. Application of the theory of planned behaviour to two dietary behaviours: roles of perceived control and self-efficacy. Br J Health Psychol. (2000) 5:121–39. 10.1348/135910700168810

[13] Allom V Mullan B. Self-regulation versus habit: the influence of self-schema on fruit and vegetable consumption. Psychol Health. (2012) 27:7–24. 10.1080/08870446.2011.605138 21827291

[14] Kothe EJ Mullan BA Butow P. Promoting fruit and vegetable consumption. Testing an intervention based on the theory of planned behavior. Appetite. (2012) 58:997–1004. 10.1016/j.appet.2012.02.012 22349778

[15] Menozzi D Sogari G Mora C. Explaining vegetable consumption among young adults: an application of the theory of planned behaviour. Nutrients. (2015) 7:7633–50. 10.3390/nu7095357 26378570 PMC4586552

[16] Cunha DB Souza B da SN de Pereira RA Sichieri R. Effectiveness of a randomized school-based intervention involving families and teachers to prevent excessive weight gain among adolescents in Brazil. PLoS ONE. (2013) 8:e0057498. 10.1371/journal.pone.0057498 23451237 PMC3581462

[17] Toral N Slater B. Intervention based exclusively on stage-matched printed educational materials regarding healthy eating does not result in changes to adolescents' dietary behavior. Sci World J. (2012) 2012:174640. 10.1100/2012/174640 22545007 PMC3322369

[18] Kokanović A Mandić M Banjari I. Does individual dietary intervention have any impact on adolescents with cardiovascular health risks. Med Glas (Zenica). (2014) 11:234–7. 24496370

[19] Yusoff H Wan Daud WN Ahmad Z. Effectiveness of nutrition education vs. non-nutrition education intervention in improving awareness pertaining iron deficiency among anemic adolescents. Iran J Public Health. (2013) 42:467–71. 23802103 PMC3684454

[20] Leme ACB Philippi ST. Cultural adaptation and psychometric properties of social cognitive scales related to adolescent dietary behaviors. Cad Saúde Coletiva. (2014) 22:252–9. 10.1590/1414-462X201400030006

[21] Tucunduva Philippi S Guerra PH Barco Leme AC. Health behavioral theories used to explain dietary behaviors in adolescents: a systematic review. Nutrire. (2016) 41:22. 10.1186/s41110-016-0023-9

[22] Bandura A. Social Foundations of Thought and Action. Hobokem, NJ: Prentice-Hall (1986).

[23] Herman CP Roth DA Polivy J. Effects of the presence of others on food intake: a normative interpretation. Psychol Bull. (2003) 129:873–86. 10.1037/0033-2909.129.6.873 14599286

[24] Fishbein M AJZEN I. Predicting and Changing Behavior: The reason action approach, 1st edn. New York, NY: Psychology Press - Taylor & Francis Group (2010).

[25] McEachan R Taylor N Harrison R Lawton R Gardner P Conner M. Meta-analysis of the reasoned action approach (RAA) to understanding health behaviors. Ann Behav Med. (2016) 50:592–612. 10.1007/s12160-016-9798-4 27169555 PMC4933736

[26] Steinmetz H Knappstein M Ajzen I Schmidt P Kabst R. How effective are behavior change interventions based on the theory of planned behavior? Zeitschrift für Psychologie. (2016) 224:216–33. 10.1027/2151-2604/a000255

[27] Ajzen I. Attitudes, Personality and Behavior. Milton Keynes: Open University Press (1988).

[28] Ajzen I. The theory of planned behavior. Org Behav Hum Decis Process. (1991) 50:179–211. 10.1016/0749-5978(91)90020-T

[29] Ajzen I Fishbein M. Understanding Attitudes and Predicting Social Behavior. Hoboken, NJ: Prentice-Hall (1980).

[30] AJZEN I. Icek Ajzen. Freq Asked Quest What is Differ Between Perceived Behav Control self-efficacy? (2020). Available online at: http://people.umass.edu/aizen/faq.html (accessed July 11, 2020).

[31] Fishbein M. A reasoned action approach to health promotion. Med Decis Mak. (2008) 28:834–44. 10.1177/0272989X08326092 19015289 PMC2603050

[32] Eagly AH Chaiken S. The Psychology of Attitudes. New York, NY: Harcourt Brace Jovanovich College Publishers (1993).

[33] Ajzen I Dasgupta N. Explicit and Implicit Beliefs, Attitudes, and Intentions: the Role of Conscious and Unconscious Processes in Human Behavior. Oxford: Oxford University Press. (2015). 10.1093/acprof:oso/9780190267278.003.0005

[34] Higgs S. Social norms and their influence on eating behaviours. Appetite. (2015) 86:38–44. 10.1016/j.appet.2014.10.021 25451578

[35] Roth D Herman C Polivy J Pliner P. Self-presentational conflict in social eating situations: a normative perspective. Appetite. (2001) 36:165–71. 10.1006/appe.2000.0388 11237352

[36] Cialdini RB Goldstein NJ. Social influence: compliance and conformity. Annu Rev Psychol. (2004) 55:591–621. 10.1146/annurev.psych.55.090902.142015 14744228

[37] Povey R Conner M Sparks P James R Shepherd R. The theory of planned behaviour and healthy eating: examining additive and moderating effects of social influence variables. Psychol Health. (2000) 14:991–1006. 10.1080/08870440008407363 22175258

[38] Cox DN Anderson AS Lean ME Mela DJ UK consumer attitudes, beliefs and barriers to increasing fruit and vegetable consumption. Public Health Nutr. (1998) 1:61–8. 10.1079/PHN19980009 10555532

[39] Kothe EJ Mullan BA. Interaction effects in the theory of planned behaviour: predicting fruit and vegetable consumption in three prospective cohorts. Br J Health Psychol. (2015) 20:549–62. 10.1111/bjhp.12115 25209256

[40] Pandey S Budhathoki M Yadav DK. Psychosocial determinants of vegetable intake among nepalese young adults: an exploratory survey. Front Nutr. (2021) 8:688059 10.3389/fnut.2021.688059 34179061 PMC8222569

[41] Hartman H Wadsworth DP Penny S van Assema P Page R. Psychosocial determinants of fruit and vegetable consumption among students in a New Zealand university. Results of focus group interviews. Appetite. (2013) 65:35–42. 10.1016/j.appet.2013.02.005 23415984

[42] Sato Y Miyanaga M Wang DH. Psychosocial determinants of fruit and vegetable intake in japanese adolescents: a school-based study in japan. Int J Environ Res Public Health. (2020) 17:1–11. 10.3390/ijerph17155550 32751998 PMC7432351

[43] Berg C Jonsson I M C. Understanding choice of milk and bread for breakfast among Swedish children aged 11–15 years: an application of the Theory of Planned Behaviour. Appetite. (2000) 34:5–19. 10.1006/appe.1999.0269 10744887

[44] Nunes LP Dutra FM Borges JAR. Consumo de peixes: uma aplicação da teoria do comportamento planejado. Rev Bras Adm Científica. (2020) 11:189–204. 10.6008/CBPC2179-684X.2020.001.0014

[45] Forero CG Maydeu-Olivares A Gallardo-Pujol D. Factor analysis with ordinal indicators: a monte carlo study comparing DWLS and ULS estimation. Struct Equ Model A Multidiscip J. (2009) 16:625–41. 10.1080/10705510903203573

[46] de Jesus Mendes da Fonseca M Faerstein E Chor D Lopes CS. Validade de peso e estatura informados e índice de massa corporal: Estudo pró-saúde. Rev Saude Publica. (2004) 38:392–8. 10.1590/S0034-89102004000300009 15243669

[47] Klipin M Mare I Hazelhurst S Kramer B. The process of installing REDCap, a web based database supporting biomedical research: the first year. Appl Clin Inform. (2014) 5:916–29. 10.4338/ACI-2014-06-CR-0054 25589907 PMC4287671

[48] R Core Team. A language and Environment for Statistical Computing. Vienna: R Core Team (2013).

[49] JASP team,. JASP. (2021). Available online at: https://jasp-stats.org/ (accessed September 20, 2021).

[50] Morris TP White IR Royston P. Tuning multiple imputation by predictive mean matching and local residual draws. BMC Med Res Methodol. (2014) 14:1–13. 10.1186/1471-2288-14-75 24903709 PMC4051964

[51] Schafer JL Olsen MK. Multiple imputation for multivariate missing-data problems: a data analyst's perspective. Multivariate Behav Res. (1998) 33:545–71. 10.1207/s15327906mbr3304_5 26753828

[52] Hair JF Jr Black WC Babin BJ Anderson RE. Multivariate Data Analysis, 7th edn. Upper Saddle River: Prentice Hall (2010).

[53] Bagozzi RP Yi Y. Specification, evaluation, and interpretation of structural equation models. J Acad Mark Sci. (2012) 40:8–34. 10.1007/s11747-011-0278-x

[54] Schreiber J Nora A. Reporting structural equation modeling and confirmatory factor analysis results: a review. J Educ Res. (2006) 6:323–38. 10.3200/JOER.99.6.323-338

[55] Marôco J. Análise de Equações Estruturais: fundamentos teóricos, software & aplicações. 2 ed. Pêro Pinheiro: Soluções Gráficas (2014).

[56] Watkins MW. The reliability of multidimensional neuropsychological measures : from alpha to omega. Clin Neuropsychol. (2017) 31:1–14. 10.1080/13854046.2017.1317364 28429633

[57] Fornell C Larcker DF Fornell C Larcker DF. Evaluating structural equation models with unobservable variables and.pdf. J Mark Res. (1981) XVIII:39–50. 10.1177/002224378101800104

[58] Henseler J Ringle CM Sarstedt M. A new criterion for assessing discriminant validity in variance-based structural equation modeling. J Acad Mark Sci. (2015) 43:115–35. 10.1007/s11747-014-0403-8

[59] Leal GVdS Philippi ST Matsudo SMM Toassa EC. Consumo alimentar e padrão de refeições de adolescentes, São Paulo, Brasil Food intake and meal patterns of adolescents, São Paulo, Brazil. Rev Bras Epidemiol. (2010) 13:457–67. 10.1590/S1415-790X2010000300009 20857032

[60] Blanchard CM Fisher J Sparling PB Shanks TH Nehl E Rhodes RE. Understanding adherence to 5 servings of fruits and vegetables per day: a theory of planned behavior perspective. J Nutr Educ Behav. (2009) 41:3–10. 10.1016/j.jneb.2007.12.006 19161914 PMC8190952

[61] John DR. Consumer socialization of children: a retrospective look at twenty-five years of research. J Consum Res. (1999) 26:183–213. 10.1086/209559

[62] Avery A Anderson C McCullough F. Associations between children's diet quality and watching television during meal or snack consumption: a systematic review. Matern Child Nutr. (2017) 13:12428. 10.1111/mcn.12428 28211230 PMC6866147

[63] Lipsky LM Iannotti RJ. Associations of television viewing with eating behaviors in the 2009 health behaviour in school-aged children study. Arch Pediatr Adolesc Med. (2012) 166:465–72. 10.1001/archpediatrics.2011.1407 22566548 PMC4733642

[64] Chapman CD Nilsson VC Thune HA Cedernaes J Le Greves M Hogenkamp PS. Watching TV and food intake: the role of content. PLoS ONE. (2014) 9:e100602. 10.1371/journal.pone.0100602 24983245 PMC4077693

[65] Ares G Machín L Girona A Curutchet MR Giménez A. Uma comparação dos motivos subjacentes às escolhas alimentares e das barreiras contra a alimentação saudável entre consumidores de renda baixa e média no Uruguai. Cad Saude Publica. (2017) 33:1–12. 10.1590/0102-311x00213315 28538799

[66] Garvey W Mechanick J. Proposal for a scientifically correct and medically actionable disease classification system (ICD) for obesity. Obesity. (2020) 28:484–92. 10.1002/oby.22727 32090513 PMC7045990

[67] Appelhans BM Whited MC Schneider KL Pagoto SL. Time to abandon the notion of personal choice in dietary counseling for obesity? J Am Diet Assoc. (2011) 111:1130–113. 10.1016/j.jada.2011.05.014 21802557 PMC3148487

[68] Wharton S Lau DCW Vallis M Sharma AM Biertho L Campbell-Scherer D. Obesity in adults: a clinical practice guideline. CMAJ. (2020) 192:E875–91. 10.1503/cmaj.191707 32753461 PMC7828878

[69] Krølner R Rasmussen M Brug J Klepp K-I Wind M Due P. Determinants of fruit and vegetable consumption among children and adolescents: a review of the literature. Part II: qualitative studies. Int J Behav Nutr Phys Act. (2011) 8:112. 10.1186/1479-5868-8-112 21999291 PMC3260149

[70] Sharps M Robinson E. Encouraging children to eat more fruit and vegetables: Health vs. descriptive social norm-based messages. Appetite. (2016) 100:18–25. 10.1016/j.appet.2016.01.031 26820776 PMC4819560

[71] Robinson E Fleming A Higgs S. Prompting healthier eating: testing the use of health and social norm based messages. Health Psychol. (2014) 33:1057–64. 10.1037/a0034213 24295025

[72] Higgs S Liu J Collins EIM Thomas JM. Using social norms to encourage healthier eating. Nutr Bull. (2019) 44:43–52. 10.1111/nbu.1237126072177

[73] Luszczynska A Horodyska K Zarychta K Liszewska N Knoll N Scholz U. Planning and self-efficacy interventions encouraging replacing energy-dense foods intake with fruit and vegetable: a longitudinal experimental study. Psychol Heal. (2016) 31:40–64. 10.1080/08870446.2015.1070156 26160226

