# Supplementary material for: Exploring Psychosocial Determinants of Eating Behavior: Fruit and Vegetable Intake Among Brazilian Adolescents
Source: Front Nutr. 2021 Dec 16;8:796894. doi: 10.3389/fnut.2021.796894 (PMC8716615; doi:10.3389/fnut.2021.796894)
Supplement: Supplementary file 1 [file Table_1.DOCX]

Supplementary Material

**Tabel 1.** Skewness and Kurtosis for observed variables from week frequency of FV, social psychological determinants, SES and BMI.

| **Item** | **Skewness** | **Std. Error of Skewness** | **Kurtosis** | **Std. Error of Kurtosis** |
| --- | --- | --- | --- | --- |
| **Freq1** | 1.457 | 0.118 | 1.757 | 0.235 |
| **Freq2** | 1.687 | 0.118 | 2.594 | 0.235 |
| **Freq3** | 1.206 | 0.118 | 0.687 | 0.235 |
| **Self1** | -0.586 | 0.118 | -0.672 | 0.235 |
| **Self2** | -0.368 | 0.118 | -0.829 | 0.235 |
| **Self3** | -0.103 | 0.118 | -1.109 | 0.235 |
| **Self4** | 0.120 | 0.118 | -1.226 | 0.235 |
| **Self5** | 0.156 | 0.118 | -1.003 | 0.235 |
| **Self6** | 0.652 | 0.118 | -0.611 | 0.235 |
| **Self7** | 0.606 | 0.118 | -0.558 | 0.235 |
| **Self8** | 0.245 | 0.118 | -1.023 | 0.235 |
| **At1** | -0.264 | 0.118 | -1.016 | 0.235 |
| **At2** | -0.655 | 0.118 | -0.544 | 0.235 |
| **At3** | -1.195 | 0.118 | 0.731 | 0.235 |
| **At4** | -0.626 | 0.118 | -0.630 | 0.235 |
| **At5** | -0.066 | 0.118 | -1.352 | 0.235 |
| **At6** | -0.297 | 0.118 | -0.997 | 0.235 |
| **At7** | -0.722 | 0.118 | -0.431 | 0.235 |
| **At8** | -0.173 | 0.118 | -1.117 | 0.235 |
| **At9** | -0.645 | 0.118 | -0.607 | 0.235 |
| **Descript1** | -1.433 | 0.118 | 1.559 | 0.235 |
| **Descript2** | -0.963 | 0.118 | -0.216 | 0.235 |
| **Descript3** | -0.032 | 0.118 | -0.716 | 0.235 |
| **Descript4** | 0.344 | 0.118 | -1.161 | 0.235 |
| **Injunctive1** | -1.491 | 0.118 | 1.547 | 0.235 |
| **Injunctive2** | -1.107 | 0.118 | 0.150 | 0.235 |
| **Injunctive3** | 0.371 | 0.118 | -0.883 | 0.235 |
| **Injunctive4** | -0.327 | 0.118 | -1.059 | 0.235 |
| **BMI** | 1.011 | 0.118 | 1.478 | 0.235 |
| **SES** | 0.453 | 0.118 | 1.679 | 0.235 |

**Table 2.** Psychossocial Influences for fruit and vegetable Eating Scale - PSI-FAVES.

| Report of Week frequency of fruit and vegetables (FV) | Never  (1) | Once a week  (2) | Twice a week (3) |  | Three times a week  (4) | Four times a week  (5) | Five times a week  (6) | Six Times a Week  (7) | Seven times a week  (8) |
| --- | --- | --- | --- | --- | --- | --- | --- | --- | --- |
| Fr1: “How many times do you eat fruits in your breakfast” | ( ) | ( ) | ( ) |  | ( ) | ( ) | ( ) | ( ) | ( ) |
| Fr2: “How many times do you eat fruits in your lunch” | ( ) | ( ) | ( ) |  | ( ) | ( ) | ( ) | ( ) | ( ) |
| Fr3: “how many times do you eat vegetables in your intervals (“e.g., sandwich with thin carrots, lettuce or tomatoes for a morning or afternoon snack)” | ( ) | ( ) | ( ) |  | ( ) | ( ) | ( ) | ( ) | ( ) |

| Self-efficacy | Not sure at all  (1) | Not too sure  (2) | Sure  (3) |  | Very Sure  (4) | Completely sure  (5) |
| --- | --- | --- | --- | --- | --- | --- |
| Self1: “I feel able to eat more fruit and vegetables everyday” | ( ) | ( ) | ( ) |  | ( ) | ( ) |
| Self2: “I fell able to eat more fruit and vegetables when I get home from school or work” | ( ) | ( ) | ( ) |  | ( ) | ( ) |
| Self3: “I feel able to eat more fruit and vegetables while watching TV” | ( ) | ( ) | ( ) |  | ( ) | ( ) |
| Self4: “I fell able to eat more fruit and vegetable while using computer or cellphone” | ( ) | ( ) | ( ) |  | ( ) | ( ) |
| Self5: “I feel able to eat fruit and vegetable when my friends are around” | ( ) | ( ) | ( ) |  | ( ) | ( ) |
| Self6: “I feel able to eat fruit and vegetable when I´m bored” | ( ) | ( ) | ( ) |  | ( ) | ( ) |
| Self7: “I feel able to eat fruit and vegetable when I´m in a bad mood” | ( ) | ( ) | ( ) |  | ( ) | ( ) |
| Self8: “I feel able to eat fruit and vegetables when I´m busy” | ( ) | ( ) | ( ) |  | ( ) | ( ) |

| *Attitudes* | Totally disagree  (1) | Disagree  (2) | Agree  (3) | Totally agree  (4) | Strongly agree  (5) |
| --- | --- | --- | --- | --- | --- |
| At1: “If I eat fruits and vegetables I´ll like myself better” | ( ) | ( ) | ( ) | ( ) | ( ) |
| At2: “If I eat fruit and vegetables I´ll lose weight” | ( ) | ( ) | ( ) | ( ) | ( ) |
| At3: “If I eat fruits and vegetables I´ll look better (e.g. my skin, hair and nails will look better)” | ( ) | ( ) | ( ) | ( ) | ( ) |
| At4: “If I eat fruits and vegetables I´ll look better (e.g. my skin, hair and nails will look better) and I´ll be more confident with myself when my friends are around” | ( ) | ( ) | ( ) | ( ) | ( ) |
| At5: “If I eat fruits and vegetables I will look better (eg my skin, hair and nails will look better) and I would like to show this improvement by posting texts and/or photos on social media (Facebook®, Instagram®, Periscope®, Snapchat®, Blogs, etc) | ( ) | ( ) | ( ) | ( ) | ( ) |
| At6: “If I eat more fruits and vegetables my family will also eat more” | ( ) | ( ) | ( ) | ( ) | ( ) |
| At7: "If I eat more fruits and vegetables my family will be proud of me" | ( ) | ( ) | ( ) | ( ) | ( ) |
| At8: “If I eat more fruits and vegetables I will also influence my friends to eat more” | ( ) | ( ) | ( ) | ( ) | ( ) |
| At9: “If I eat more fruits and vegetables I will be an example of health to my friends” | ( ) | ( ) | ( ) | ( ) | ( ) |

| *Descriptive Norms* “Please think about people you know eat” | Don´t Know  (1) | Totally disagree  (2) | Disagree  (3) |  | Agree  (4) | Totally agree  (5) |
| --- | --- | --- | --- | --- | --- | --- |
| Descriptive1: “My mother eats fruits and vegetables” | ( ) | ( ) | ( ) |  | ( ) | ( ) |
| Descriptive2: “My father eats fruits and vegetables” | ( ) | ( ) | ( ) |  | ( ) | ( ) |
| Descriptive3: “My friends eat fruits and vegetables” | ( ) | ( ) | ( ) |  | ( ) | ( ) |
| Descriptive4: “People I follow on social media (Facebook, Instagram, Periscope, Snapchat, Blogs) post photos eating fruits and vegetables because they seem to care about their health” | ( ) | ( ) | ( ) |  | ( ) | ( ) |

| *Injunctive norms* “Please think about people you know eat” | Don´t Know  (1) | Totally disagree  (2) | Disagree  (3) |  | Agree  (4) | Totally agree  (5) |
| --- | --- | --- | --- | --- | --- | --- |
| Injunctive1: *“My mother thinks I should eat more fruits and vegetables”* | ( ) | ( ) | ( ) |  | ( ) | ( ) |
| Injunctive2: “*My father thinks I should eat more fruits and vegetables”* | ( ) | ( ) | ( ) |  | ( ) | ( ) |
| Injunctive3: “*My friends think I should eat more fruits and vegetables”* | ( ) | ( ) | ( ) |  | ( ) | ( ) |
| Injunctive4: *"People I follow on social networks (Facebook, Instagram, Periscope, Snapchat, Blogs) argue that healthy habits like eating fruits and vegetables are important things and that's why I think I should think and do the same”* | ( ) | ( ) | ( ) |  | ( ) | ( ) |

**Table 3.** Pearson's Correlations for items of PSY-FAVES

| **Item** | **1** | **2** | **3** | **4** | **5** | **6** | **7** | **8** | **9** | **10** | **11** | **12** | **13** | **14** | **15** | **16** | **17** | **18** | **19** | **20** | **21** | **22** | **23** | **24** | **25** | **26** | **27** | **28** |
| --- | --- | --- | --- | --- | --- | --- | --- | --- | --- | --- | --- | --- | --- | --- | --- | --- | --- | --- | --- | --- | --- | --- | --- | --- | --- | --- | --- | --- |
| 1. Freq1 | — |  |  |  |  |  |  |  |  |  |  |  |  |  |  |  |  |  |  |  |  |  |  |  |  |  |  |  |
| 2. Freq2 | 0.288 | — |  |  |  |  |  |  |  |  |  |  |  |  |  |  |  |  |  |  |  |  |  |  |  |  |  |  |
| 3. Freq7 | 0.309 | 0.19 | — |  |  |  |  |  |  |  |  |  |  |  |  |  |  |  |  |  |  |  |  |  |  |  |  |  |
| 4. Self1 | 0.236 | 0.218 | 0.278 | — |  |  |  |  |  |  |  |  |  |  |  |  |  |  |  |  |  |  |  |  |  |  |  |  |
| 5. Self2 | 0.193 | 0.25 | 0.281 | 0.58 | — |  |  |  |  |  |  |  |  |  |  |  |  |  |  |  |  |  |  |  |  |  |  |  |
| 6. Self3 | 0.157 | 0.184 | 0.182 | 0.382 | 0.427 | — |  |  |  |  |  |  |  |  |  |  |  |  |  |  |  |  |  |  |  |  |  |  |
| 7. Self4 | 0.069 | 0.099 | 0.056 | 0.31 | 0.361 | 0.542 | — |  |  |  |  |  |  |  |  |  |  |  |  |  |  |  |  |  |  |  |  |  |
| 8. Self5 | 0.136 | 0.197 | 0.162 | 0.434 | 0.427 | 0.394 | 0.402 | — |  |  |  |  |  |  |  |  |  |  |  |  |  |  |  |  |  |  |  |  |
| 9. Self6 | 0.177 | 0.195 | 0.16 | 0.301 | 0.337 | 0.284 | 0.249 | 0.394 | — |  |  |  |  |  |  |  |  |  |  |  |  |  |  |  |  |  |  |  |
| 10. Self7 | 0.188 | 0.224 | 0.166 | 0.324 | 0.331 | 0.36 | 0.278 | 0.434 | 0.699 | — |  |  |  |  |  |  |  |  |  |  |  |  |  |  |  |  |  |  |
| 11. Self9 | 0.171 | 0.208 | 0.173 | 0.361 | 0.323 | 0.306 | 0.324 | 0.407 | 0.304 | 0.383 | — |  |  |  |  |  |  |  |  |  |  |  |  |  |  |  |  |  |
| 12. At2 | 0.12 | 0.142 | 0.177 | 0.256 | 0.27 | 0.226 | 0.127 | 0.281 | 0.152 | 0.202 | 0.338 | — |  |  |  |  |  |  |  |  |  |  |  |  |  |  |  |  |
| 13. At4 | 0.032 | 0.101 | 0.088 | 0.138 | 0.153 | 0.117 | 0.105 | 0.148 | 0.055 | 0.056 | 0.089 | 0.363 | — |  |  |  |  |  |  |  |  |  |  |  |  |  |  |  |
| 14. At5 | 0.006 | 0.07 | 0.06 | 0.167 | 0.199 | 0.174 | 0.147 | 0.24 | 0.055 | 0.067 | 0.103 | 0.362 | 0.253 | — |  |  |  |  |  |  |  |  |  |  |  |  |  |  |
| 15. At6 | 0.004 | 0.154 | 0.049 | 0.172 | 0.185 | 0.164 | 0.174 | 0.28 | 0.16 | 0.146 | 0.19 | 0.505 | 0.293 | 0.585 | — |  |  |  |  |  |  |  |  |  |  |  |  |  |
| 16. At7 | 0.038 | 0.105 | 0.1 | 0.06 | 0.159 | 0.114 | 0.131 | 0.198 | 0.173 | 0.113 | 0.136 | 0.43 | 0.285 | 0.34 | 0.5 | — |  |  |  |  |  |  |  |  |  |  |  |  |
| 17. At8 | 0.083 | 0.068 | 0.064 | 0.242 | 0.224 | 0.165 | 0.219 | 0.268 | 0.292 | 0.272 | 0.223 | 0.356 | 0.201 | 0.205 | 0.328 | 0.253 | — |  |  |  |  |  |  |  |  |  |  |  |
| 18. At9 | -0.019 | 0.111 | 0.053 | 0.15 | 0.138 | 0.19 | 0.145 | 0.239 | 0.163 | 0.151 | 0.226 | 0.391 | 0.253 | 0.288 | 0.377 | 0.297 | 0.36 | — |  |  |  |  |  |  |  |  |  |  |
| 19. At10 | 0.176 | 0.123 | 0.063 | 0.266 | 0.281 | 0.259 | 0.184 | 0.366 | 0.227 | 0.256 | 0.217 | 0.371 | 0.156 | 0.192 | 0.348 | 0.323 | 0.425 | 0.406 | — |  |  |  |  |  |  |  |  |  |
| 20. At11 | 0.127 | 0.117 | 0.03 | 0.209 | 0.182 | 0.173 | 0.107 | 0.19 | 0.193 | 0.196 | 0.162 | 0.348 | 0.151 | 0.232 | 0.308 | 0.228 | 0.311 | 0.41 | 0.496 | — |  |  |  |  |  |  |  |  |
| 21. Descript1 | 0.086 | 0.073 | 0.097 | 0.198 | 0.206 | 0.166 | 0.11 | 0.182 | 0.118 | 0.175 | 0.007 | 0.09 | 0.135 | 0.112 | 0.095 | 0.069 | 0.114 | 0.169 | 0.141 | 0.166 | — |  |  |  |  |  |  |  |
| 22. Descript2 | 0.081 | 0.079 | 0.14 | 0.072 | 0.139 | 0.19 | 0.2 | 0.161 | 0.059 | 0.118 | 0.126 | 0.097 | 0.059 | 0.088 | 0.043 | 0.105 | 0.11 | 0.165 | 0.1 | 0.118 | 0.428 | — |  |  |  |  |  |  |
| 23. Descript3 | 0.083 | 0.04 | 0.055 | 0.225 | 0.177 | 0.217 | 0.178 | 0.226 | 0.151 | 0.153 | 0.125 | 0.154 | 0.072 | 0.089 | 0.158 | 0.128 | 0.19 | 0.178 | 0.154 | 0.106 | 0.229 | 0.241 | — |  |  |  |  |  |
| 24. Descript4 | 0.1 | 0.046 | 0.093 | 0.096 | 0.125 | 0.174 | 0.164 | 0.129 | 0.109 | 0.065 | 0.068 | 0.116 | 0.047 | 0.052 | 0.044 | 0.203 | 0.078 | 0.023 | 0.173 | 0.109 | 0.052 | 0.129 | 0.251 | — |  |  |  |  |
| 25. Injunctive1 | -0.075 | -0.039 | -0.014 | -0.073 | 0.008 | 0.009 | -0.006 | -0.017 | 0.042 | 0.029 | 0.032 | 0.155 | 0.154 | 0.221 | 0.245 | 0.154 | 0.214 | 0.298 | 0.138 | 0.233 | 0.271 | 0.114 | 0.065 | -0.033 | — |  |  |  |
| 26. Injunctive2 | -0.044 | 0.032 | 0.04 | -0.011 | 0.038 | 0.079 | 0.041 | 0.013 | 0.036 | 0.058 | 0.115 | 0.182 | 0.138 | 0.208 | 0.214 | 0.157 | 0.177 | 0.273 | 0.154 | 0.205 | 0.139 | 0.427 | 0.066 | 0.026 | 0.618 | — |  |  |
| 27. Injunctive3 | 0.069 | 0.04 | 0.026 | 0.195 | 0.205 | 0.164 | 0.123 | 0.199 | 0.237 | 0.259 | 0.211 | 0.255 | 0.131 | 0.165 | 0.224 | 0.246 | 0.292 | 0.262 | 0.376 | 0.287 | 0.1 | 0.088 | 0.328 | 0.214 | 0.32 | 0.304 | — |  |
| 28. Injunctive4 | 0.065 | 0.054 | 0.041 | 0.175 | 0.123 | 0.231 | 0.184 | 0.195 | 0.189 | 0.179 | 0.246 | 0.234 | 0.049 | 0.096 | 0.193 | 0.22 | 0.14 | 0.224 | 0.249 | 0.193 | 0.094 | 0.153 | 0.212 | 0.509 | 0.18 | 0.201 | 0.294 | — |

**Table 4.** Hetero-trait mono-trait analysis (HTMT) for PSY-FAVES factors.

| HTMT | 1 | 2 | 3 | 4 | 5 |
| --- | --- | --- | --- | --- | --- |
| 1.FV ^a^ | — |  |  |  |  |
| 2. Self-Efficacy | 0.57 | — |  |  |  |
| 3. Attitudes | 0.28 | 0.52 | — |  |  |
| 4. Descriptive Social Norms | 0.34 | 0.50 | 0.42 | — |  |
| 5. Injunctive Social Norms | 0.06 | 0.31 | 0.63 | 0.65 | — |

^a^ Report of Week frequency of fruit and vegetables.
